# Compromised steady‐state germinal center activity with age in nonhuman primates

**DOI:** 10.1111/acel.13087

**Published:** 2019-12-15

**Authors:** Kimberly Shankwitz, Suresh Pallikkuth, Tirupataiah Sirupangi, Daniel Kirk Kvistad, Kyle Blaine Russel, Rajendra Pahwa, Lucio Gama, Richard A. Koup, Li Pan, Francois Villinger, Savita Pahwa, Constantinos Petrovas

**Affiliations:** ^1^ Tissue Analysis Core Immunology Laboratory Vaccine Research Center NIAID NIH Bethesda MD USA; ^2^ New Iberia Research Center University of Louisiana at Lafayette Lafayette LA USA; ^3^ Microbiology and Immunology University of Miami Miller School Medicine Miami FL USA; ^4^ Department of Molecular and Comparative Pathobiology Johns Hopkins School of Medicine Baltimore USA; ^5^ Vaccine Research Center NIAID NIH Bethesda MD USA; ^6^ Immunology Laboratory Vaccine Research Center NIAID NIH Bethesda MD USA

**Keywords:** aging, B cells, follicles, Tfh cells

## Abstract

Age‐related reductions in vaccine‐induced B cells in aging indicate that germinal centers (GCs), the anatomical site where the development of humoral responses takes place, may lose efficacy with age. We have investigated the baseline follicular and GC composition in nonhuman primates (NHPs) with respect to their age. There was a marked reduction in follicular area in old animals. We found significantly lower normalized numbers of follicular PD1^hi^ CD4 T (Tfh) and proliferating (Ki67^hi^) GC B cells with aging, a profile associated with significantly higher numbers of potential follicular suppressor FoxP3^hi^Lag3^hi^ CD4 T cells. Furthermore, a positive correlation was found between Tfh and follicular CD8 T cells (fCD8) only in young animals. Despite the increased levels of circulating preinflammatory factors in aging, young animals had higher numbers of monocytes and granulocytes in the follicles, a profile negatively associated with numbers of Tfh cells. Multiple regression analysis showed an altered association between GC B cells and other GC immune cell populations in old animals suggesting a differential mechanistic regulation of GC activity in aging. Our data demonstrate defective baseline GC composition in old NHPs and provide an immunological base for further understanding the adaptive humoral responses with respect to aging.

## Graphical abstract

Steady‐state germinal center immune reactivity is compromised in aged NHPs. This loss of reactivity is characterized by changes in many GC associated cell populations including Tfh, B cells, and follicular Tregs.

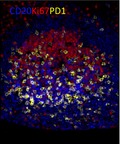

## 1 INTRODUCTION

Aging of the immune system creates many complications in elderly people that puts them at high risk for infections (Yoshikawa, 2000). The decline of the immune system can be attributed to many changes occurring within secondary lymphoid organs, the hotbeds of immune response induction, with advancing age (Gustafson, Weyand, & Goronzy, 2018; Linterman, 2014). The development of the antigen‐specific B‐cell response takes place within highly specialized areas of the lymph nodes (LNs) called follicles (Crotty, 2011). Tfh cells, found within follicles and GCs, provide critical help for B‐cell proliferation and differentiation (Crotty, 2011). The interplay between Tfh, GC B cells, and follicular dendritic cells (FDCs) in the light zone (LZ) provides the appropriate signals for the activation, proliferation, somatic hypermutation, and affinity maturation of B cells in the dark zone (DZ) (Sage, Tan, Freeman, Haigis, & Sharpe, 2015). Interrupting the cross‐talk between Tfh and B cells results in impaired GC formation and pathogen‐specific B‐cell responses in aged mice (Sage et al., 2015). Furthermore, mouse models have shown that aging impacts many of these populations including fibroblastic reticular cells (FRCs), a cellular network crucial for the survival of naïve T cells and trafficking of T cells within the extra follicular areas (Thompson, Smithey, Surh, & Nikolich‐Zugich, 2017), FDCs, vital for GC organization and antigen presentation to B cells (Aydar, Balogh, Tew, & Szakal, 2003), tissue macrophages, and Tfh and B cells (Eaton, Burns, Kusser, Randall, & Haynes, 2004; Turner & Mabbott, 2017).

Human studies have shown that loss of B cells in elderly patients is associated with weakened humoral responses (Colonna‐Romano et al., 2008). Aging of T cells affects B‐cell somatic hypermutation and output quality without affecting the number and size of follicles and GCs (Banerjee, Sanderson, Spencer, & Dunn‐Walters, 2000; Lazuardi et al., 2006). Despite reduced tonsil GC B cells (Lee et al., 2016), most elderly people can maintain the capacity to generate a diverse B‐cell repertoire (Kolar, Mehta, Wilson, & Capra, 2006). Similarly to TCR, the BCR repertoire in humans has been shown to decline with age and becomes dysfunctional (de Bourcy et al., 2016; Boyd, Liu, Wang, Martin, & Dunn‐Walters, 2013; Dunn‐Walters, 2016; Wang et al., 2014). A shift in T‐cell and B‐cell subtypes from naïve to memory, a significant increase in circulating regulatory CD4 T cells (Tregs) (Freitas et al., 2019), and changes in T‐cell and B‐cell signaling have been described in elderly individuals (Beatriz Suarez‐Alvarez, 2017; Le Page, Dupuis, Larbi, Mitwowski, & Fulop, 2018; Naylor et al., 2005). Furthermore, aging in humans is associated with increased circulating pro‐inflammatory mediators (Franceschi et al., 2018; Metcalf et al., 2017).

NHPs have been extensively used for studies in HIV/SIV, vaccine development (Sui, Gordon, Franchini, & Berzofsky, 2013), and immune aging (Cicin‐Sain et al., 2010; Messaoudi et al., 2006; Okoye et al., 2015). Here, we aimed to evaluate major GC cell populations and determine baseline differences between old and young monkeys.

## 2 RESULTS

### 2.1 Altered LN and circulating T‐cell and B‐cell dynamics in old NHPs

**Figure 1:**
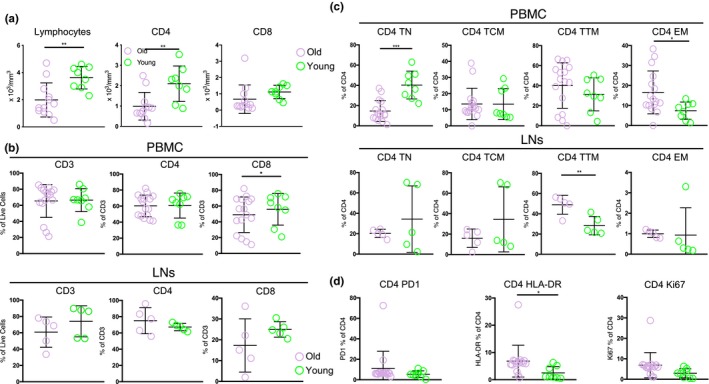
Altered immune dynamics in aging. (a) Absolute numbers of circulating total lymphocytes, CD4, and CD8 T cells in young (8) and old (16) animals. (b) Relative frequencies (flow cytometry) of circulating (upper panel, young (8) and old (16) animals) and LN‐derived (lower panel) CD3, CD4, and CD8 T cells (young (5) or old (5) animals). (c) Relative frequencies (flow cytometry) of CD4 T‐cell subsets in PBMC (young (8) and old (26) animals) and LNs (young (5) and old (5) animals). (d) Relative frequencies of circulating CD4 T cells expressing a PD‐1^hi^, HLA‐DR^hi^, or Ki67^hi^ phenotype in young (8) and old (16) animals. Student's unpaired *t* test was used. *p < .05, **p < .01, ***p < .001

First, relevant T‐cell populations in blood and LN tissues from young and old NHPs (Table 1) were analyzed. Significantly lower counts of circulating lymphocytes were found in old compared to young animals (*p* = .006) (Figure 1a), associated with a concomitant significant reduction of the absolute counts of circulating CD4 T cells (Figure 1a). No difference was found when the absolute numbers of CD8 T cells were analyzed (Figure 1a). Similar relative frequency of total CD3 and CD4 T cells in blood and LNs (Figure 1b) and a trend for lower frequency of total LN CD8 T cells (Figure 1b) in old compared to young animals were found using a polyparametric flow cytometry assay (Supporting information Figure S1a). However, a different profile for CD4 T‐cell subsets between blood and LNs with respect to age was observed. Analysis of T‐cell maturation subsets based on the expression of CD95, CD28, and CCR7 revealed a significantly lower frequency (*p* < .0001) of circulating naïve (T_N_, CD28^hi^CD95^lo^) and a significantly higher frequency (*p* = .0311) of effector memory (EM, CD28^lo^CD95^hi^) CD4 T cells (Figure 1c). A different profile was found for LN CD4 T‐cell subsets, with the transitional memory (TTM; CD28^hi^CD95^hi^CCR7^lo^) population being less frequent in young compared to old animals (*p* = .0075) (Figure 1c). As expected, the frequency of “effector” memory CD4 T cells was significantly lower in LNs compared to blood in both young (*p* = .008) and old (*p* = .004) animals (Figure 1c). A similar pattern was found when the effector CD8 T cells were analyzed in blood and LNs (Supporting information Figure S1b). A significantly higher frequency of circulating CD4 T cells expressing a HLA‐DR^hi^ profile, a surrogate of T‐cell activation, was found in old animals (*p* = .0389) (Figure 1d). However, this profile was not associated with higher levels of proliferating Ki67^hi^or PD1^hi^CD4 T cells (Figure 1d). Comparable expression of CXCR5 on circulating HLA‐DR^hi^ or PD1^hi^ CD4 T cells was found between young and old animals (Supporting information Figure S1c). Significant differences were found for several of the circulating B‐cell subsets that were analyzed, *p* = .037, 0.001, 0.035, and 0.003 for total, CD10^lo^CD21^hi^CD27^lo^IgD^hi^ naïve, CD10^lo^CD21^hi^CD27^hi^IgD^lo^ resting memory (RM), and CD10^lo^CD27^hi^ IgG‐switched memory (SW) B‐cell comparisons between young and old animals, respectively (Figure S1d). Altogether, our data indicate a significant impact of aging on several circulating and LN lymphocyte populations in NHP.

**Table 1:** Age, location, history, and assays performed for the LNs used in the study

Young animals	Age	LN/ Sampling (y)	Facility	Previous experiments	Flow cytometry	Histocytometry
A11R088	6	Inguinal (2016)	NIRC	Nanoparticle Immunization 2015	+	+
A11R091	6	Inguinal (2016)	NIRC	Nanoparticle Immunization 2015	+	+
A11R042	6	Inguinal (2016)	NIRC	Nanoparticle Immunization 2015	+	+
A11R067	5	Inguinal (2016)	NIRC	Nanoparticle Immunization 2015	+	+
A13N022	5	Axillary (2018)	NIRC	Multivalent bacterial protein immunization 2013/2016	−	+
81A	5	Axillary (2018)	JHU	No previous experimental use	−	+
50B	5	Axillary (2019)	JHU	No previous experimental use	−	+
Average	5.43					
Old animals						
95N007	21	Inguinal (2016)	NIRC	No previous experimental use	+	+
93N145	23	Inguinal (2016)	NIRC	No previous experimental use	+	+
95N139	21	Inguinal (2016)	NIRC	No previous experimental use	+	+
97N003	21	Inguinal (2016)	NIRC	No previous experimental use	+	+
ROj3	23	Axillary (2016)	YPC	Behavioral study, Listeria study	−	+
RNg2*	26	Axillary (2016)	YPC	MRI—no intervention, Listeria study, Moraten measles vaccine	−	+
ROk5*	19	Axillary (2016)	YPC	Listeria study, PET, cocaine neuropharmacology study—multiple ports were placed and PET scans, BX08 plasmid DNA IM	−	+
RUp1*	27	Axillary (2016)	YPC	Listeria study, MRIs—No intervention, In utero transplantation of human cells into primates—records are minimal, Wallen all behavioral study	−	+
Average	22.6					

* Listeria was attenuated and given intragastrically which is a noninvasive procedure. Animals were sacrificed to look for listeria in gut. Listeria was too attenuated to infect through the gut.

### 2.2 Reduced GC reactivity in old NHP LNs

**Figure 2:**
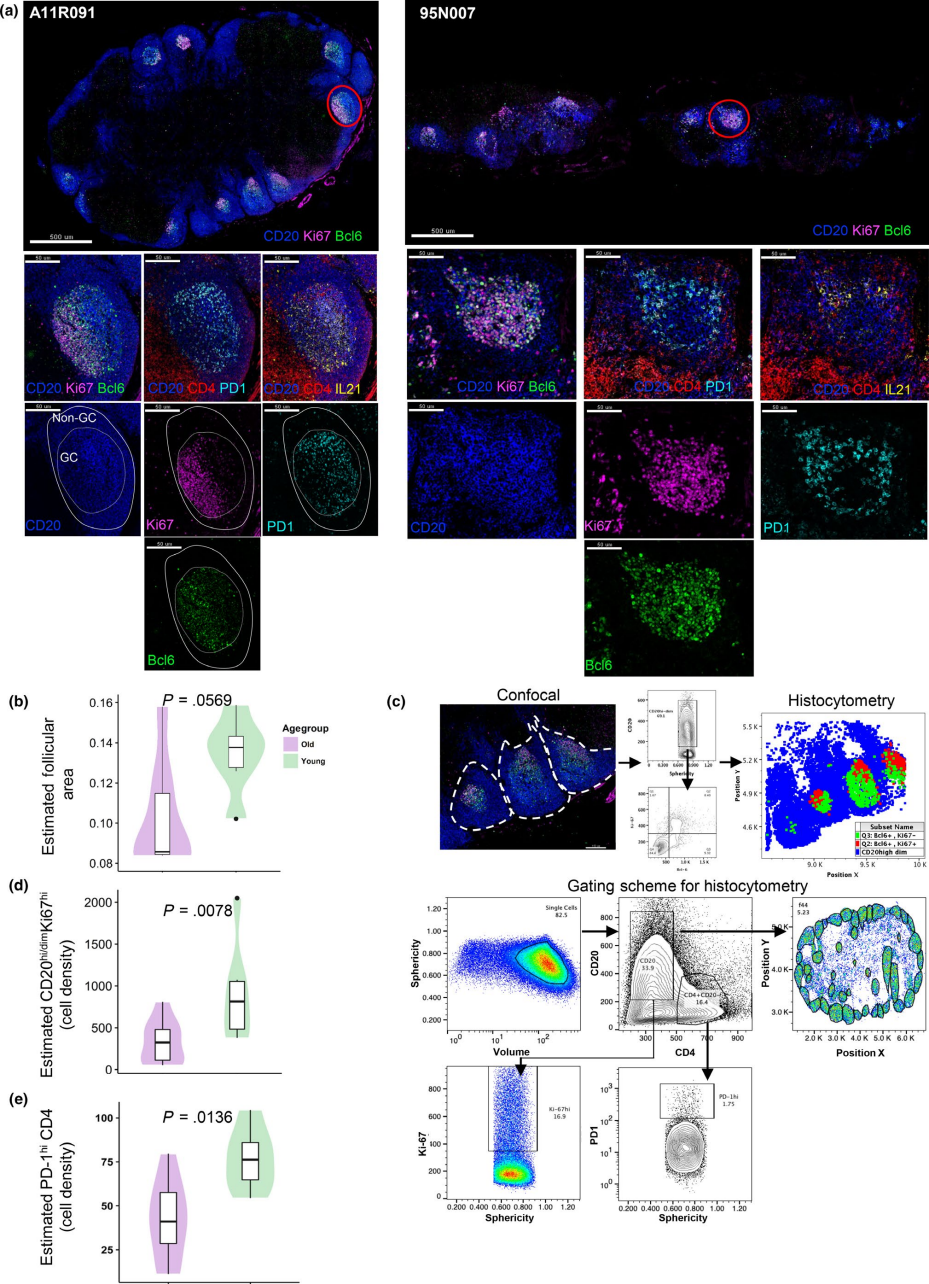
Reduced baseline germinal center (GC) reactivity in old NHPs. (a) Confocal image (40×) showing the inguinal LNs and GCs from one young NHP (A11R091, scale bar: 500 µm) and one old NHP (95N007, scale bar: 500 µm). A zoomed follicle (scale bar: 50 µm) from each animal is shown too (CD20—blue; Ki67—magenta; Bcl6—green; CD4—red; PD1—cyan; and Il‐21—yellow). The borders of a follicle (solid redline), GC (solid yellow line), and DZ (solid white line) are also shown. (b) Estimated follicular areas were calculated in young (green, 7) and old (purple, 8) animals, and a normal distribution LMM was used for analysis. (c) A confocal image (40×) of zoomed in follicles of a young NHP (A11R091) converted to histocytometry. Dotted white lines define the borders of individual follicles. The gating scheme for analysis of relevant cell populations is shown. Single cells were identified by volume and sphericity. Follicular areas were identified (CD20^hi/dim^). Analysis of CD20^hi/dim^Ki67^hi^ (d) and Tfh (e) estimated cell density (absolute numbers/ imaged area) in young (7) and old (8) animals is shown. A negative binominal GLMM was used for the analysis. Significant (<.05) *p* values are shown

Next, we performed a quantitative, multiplexed confocal imaging assay for the comparative analysis of follicular immune cell populations in LNs. Follicles were identified as CD20^hi/dim^ (Figure 2a, solid redline) while GC boundaries (Figure 2a, solid yellow line) were identified based on the expression of CD20^dim^Ki67^hi^ (abundant in the DZ, solid white line in Figure 2a) and CD20^hi^Ki67^hi/lo^ (abundant in the LZ) (Figure 2a and Figure S2a) (Ferrando‐Martinez et al., 2018). Further verification of the follicular areas was done by estimating the staining pattern of Bcl‐6, a major regulator of Tfh cell differentiation (Crotty, 2011), that is expressed by Ki67^hi^ B cells in the DZ and Ki67^lo^ B cells in the LZ (Figure 2a). GC Tfh cells were identified by staining for CD4 and PD1 as well as Bcl‐6, (Figure 2a and Figure S2a). Mature GCs were evaluated by the presence of Tfh cells and proliferating B cells and the polarization of the dark zone (where the vast majority of cells are CD20^hi/dim^Ki67^hi^) and light zone (co‐existence of Tfh and CD20 cells), which was judged by the distribution of Ki67 and Bcl6 (Figure 2a and Figure S2a). Despite the similar number of total follicles (Figure S2b), their size was found to be decreased (*p* = .0569) in old compared to young animals (Figure 2b). A trend for fewer active follicles was also found in old NHPs (Figure S2b). The polarization of GCs was less well defined in the old NHPs (Figure 2a and Figure S2a). A negative binomial generalized linear mixed‐effects model (GLMM) was used for the analysis of histocytometry (Figure 2c) generated data. Significantly higher estimated numbers of proliferating B cells (*p* = .0078) and CD4^hi^PD1^hi^ Tfh cells (*p* = .0136) per unit follicular area were found in young compared to old NHPs (Figure 2d,e and Figure S2c). Like flow cytometry (Figure 1b), histocytometry revealed a similar frequency of total LN CD4 T cells between young and old animals (Figure S2d). Interestingly, significantly higher estimated numbers of follicular CD4^hi^PD1^lo^T cells were found in old compared to young animals (Figure S2e). Further analysis of Tfh subsets with regard to the expression of Ki67 and Bcl‐6 was challenging due to the small number of identified cells. Although lower frequency of IL‐21R^hi^ Tfh cells was found in old animals (Figure S2f), no difference was found when IL‐21, a major regulator of both Tfh and B‐cell responses (Zotos et al., 2010), was investigated (Figure S2g). Contrary to bona fide GC Tfh cells, higher (*p* = .053) frequencies of circulating Tfh‐like cells (PD1^hi^CCR7^lo^CD150^lo^ICOS^hi^) within the CD4 central memory (TCM) cells in old animals were found (Figure S3a,b). Our data show a higher basal follicular/GC reactivity in young compared to old NHPs.

### 2.3 Accumulation of potential follicular suppressor FoxP3^hi^Lag3^hi^ CD4 T cells in aging

**Figure 3:**
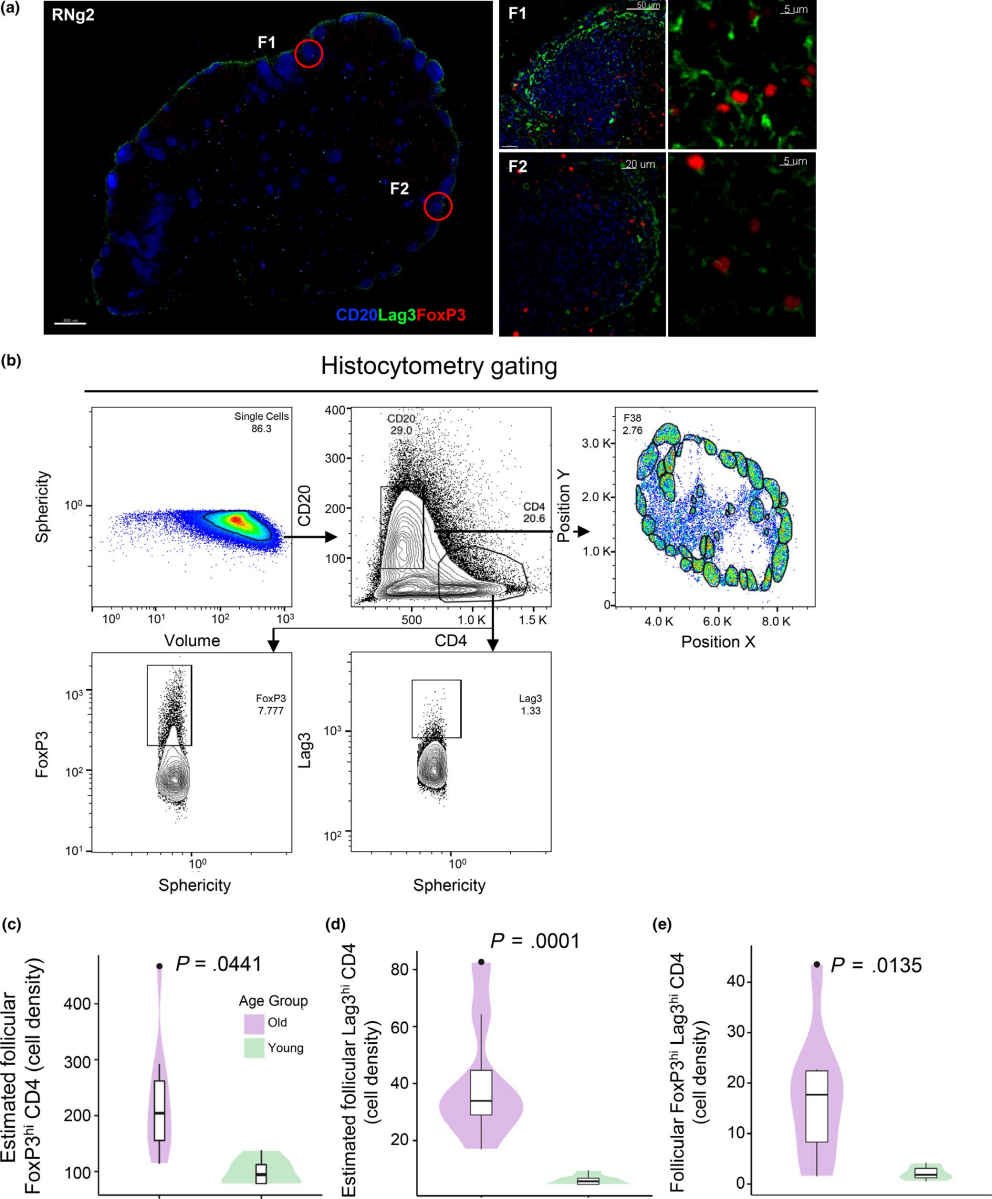
Significantly increased numbers of potential suppressor (FoxP3^hi^Lag3^hi^) CD4 T cells in aged follicles. (a) Confocal image (40×) of an inguinal LN from a young NHP (A11R088, scale bar: 500 µm) showing T regulatory cell markers. The expression of CD20 (blue), Lag3 (green), and FoxP3 (red) in two follicles and associated zoomed areas is shown (Scale bars: 50 and 5 µm). (b) The histocytometry gating scheme for the detection of relevant populations is shown. The cell density of follicular FoxP3^hi^ (c), Lag3^hi^ (d), and FoxP3^hi^Lag3^hi^ CD4 T cells (e) in young (green, 6) and old (purple, 8) animals is shown. A negative binominal GLMM was used for c and d and a two‐sample *t* test for E. Significant (<.05) *p* values are shown

Next, the expression of FoxP3 and the coinhibitory receptors Lag3 (Huan et al., 2004) and PD1 (Gianchecchi & Fierabracci, 2018) (Figure 3a,b) on CD4 T cells was analyzed. Old NHPs had significantly higher estimated numbers per unit follicular area of FoxP3^hi^ (*p* = .0441) and Lag3^hi^ (*p* = .0001) CD4 cells (Figure 3c,d) but no significant difference was found in the T‐cell zone (Figure S4a,b). We further analyzed FoxP3^hi^CD4 T cells for expression of Lag3, a receptor frequently expressed on Tregs (Huan et al., 2004) and PD‐1. Given the small number of FoxP3^hi^CD4 T cells, the average numbers for all follicular areas/per animal were used to analyze these data. Significantly increased (*p* = .0135) estimated numbers per unit follicular area of FoxP3^hi^Lag3^hi^CD4 T cells were found in old compared to young animals (Figure 3e and Figure S4c). No difference was found when the FoxP3^hi^PD‐1^dim^CD4 T cells were analyzed (Figure S4d). Our data show that aging is associated with increased recruitment of potential suppressor CD4 T cells in the follicular areas.

### 2.4 Increased CD3^hi^CD4^lo^ T‐cell numbers in old NHPs

**Figure 4:**
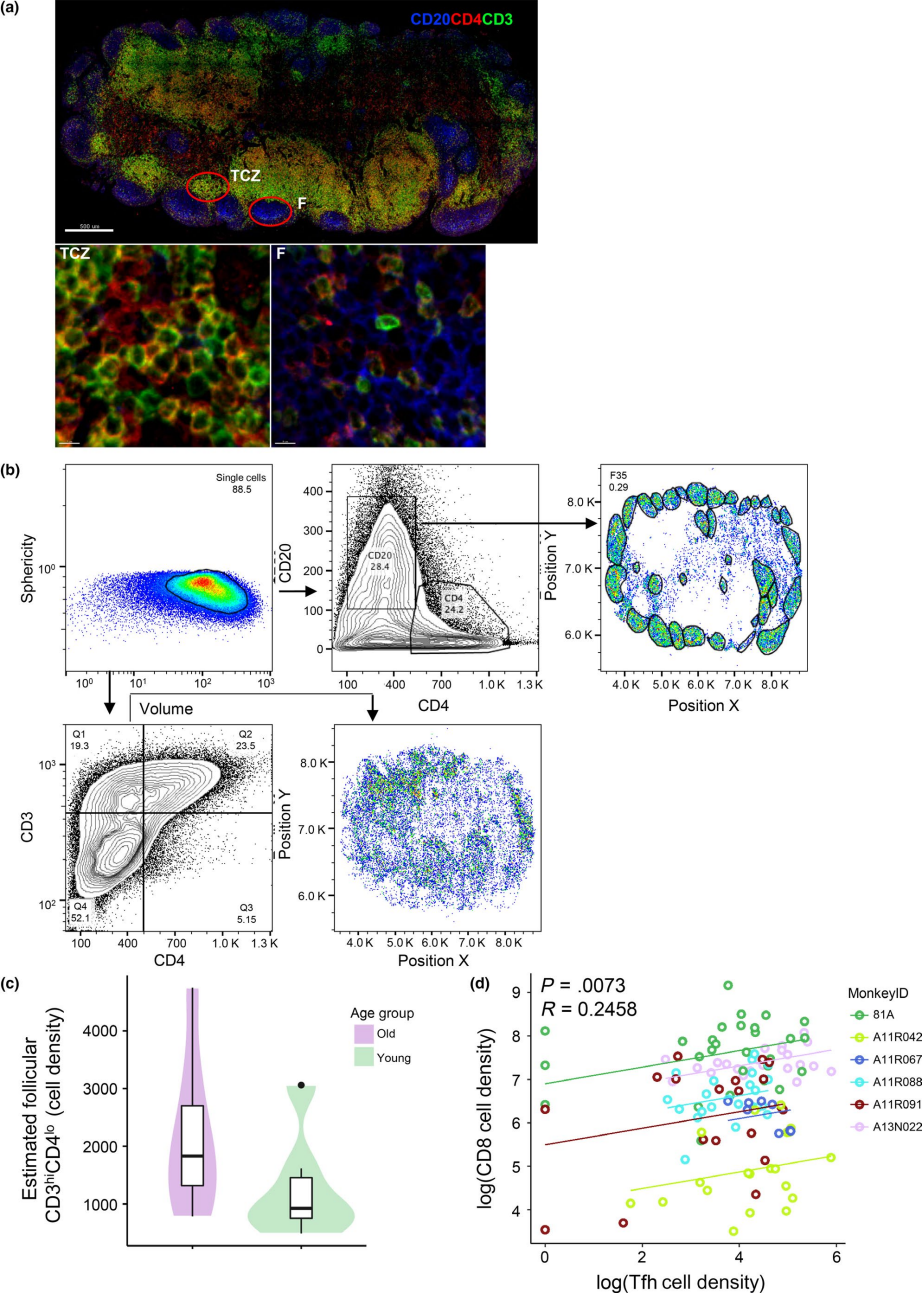
Increased numbers of CD3^hi^CD4^lo^ (CD8) T cells in aged follicles. (a) Confocal image (40×) of inguinal LN from a young NHP (A11R088, scale bar: 500 µm) showing the distribution of T cells and B cells within the LN (CD20—blue; CD4—red; and CD3—green). Zoomed areas from T‐cell zone (TCZ, scale bar: 7 µm) and a follicle (F, scale bar: 8 µm) with CD3^hi^CD4^lo^‐green and CD3^hi^CD4^hi^‐yellow. (b) The histocytometry gating scheme used to identify CD8 T cells. (c) The estimated cell density of follicular CD8 T cells in young (green, 6) and old (purple, 8) animals is shown. A GLMM via PQL was used. (d) The correlation between follicular CD8 and PD‐1^hi^ CD4 T cells in young animals is shown. Data from individual animals are shown. Each dot represents a follicle. A repeated measures correlation method was used for correlation analysis. Significant (<.05) *p* values are shown

Follicular CD8 (fCD8) T cells, potential regulators of follicular dynamics (Miles et al., 2016), accumulate during chronic viral infections (Ferrando‐Martinez et al., 2018) (Mylvaganam et al., 2017). Therefore, we sought to investigate the steady‐state dynamics of fCD8 T cells with respect to age. Given the lack of a reliable anti‐CD8 clone for FFPE NHP tissue staining, we consider the CD3^hi^CD4^lo^ T‐cell compartment to be highly enriched (the flow cytometry determined % of LN CD3^hi^CD4^lo^CD8^lo^ was 1.86 ± 0.542) in CD8 T cells (Figure 4a) as we recently described (Ferrando‐Martinez et al., 2018; Watson et al., 2018). Histocytometry analysis (Figure 4b) revealed a trend for higher, though not significant, estimated numbers per unit follicular area of CD3^hi^CD4^lo^T cells within the follicles of old compared to young animals (Figure 4c and Supporting information Figure S5a). However, no difference was found when this population was analyzed in the T‐cell zone (Figure S5b,c). Furthermore, a significant (*p* = .0073) positive correlation between CD3^hi^CD4^lo^T cells and Tfh was found selectively in young NHPs (Figure 4d).

### 2.5 Altered pro‐inflammatory LN environment between young and old NHPs

**Figure 5:**
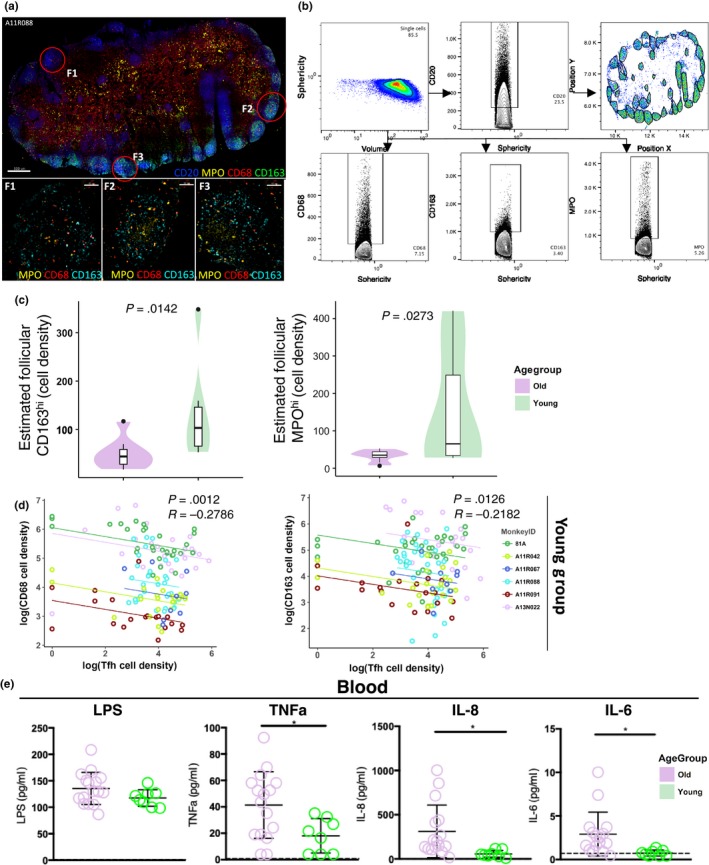
Aged follicles are characterized by reduced numbers of follicular preinflammatory cells. (a) Confocal image (40×) of an inguinal LN from a young NHP (A11R088, scale bar: 500 µm) showing the follicles and distribution of monocyte markers. Three magnified follicles (scale bar: 50 µm) are shown with MPO (yellow), CD68 (red), and CD163 (green). (b) The histocytometry gating scheme for identification of pre‐inflammatory populations is shown. (c) The follicular cell densities for CD163^hi^ and MPO^hi^ cells in young (green, 6) and old (purple, 8) are shown. A negative binomial GLMM was used. Significant (<.05) *p* values are shown. (d) Correlation analysis between follicular CD68 or CD163 and Tfh cell density in young animals. Each dot represents a follicle. A repeated measures correlation method was used for correlation analysis. Significant (<.05) *p* values are shown. (E) The levels of LPS, TNFa, IL‐8, and IL‐6 in the blood of young (8) and old (16) NHPs are shown. Each dot represents one animal. Student's unpaired *t* test was used for the analysis. *p < .05, **p < .01, ***p < .001

Tissue inflammation could represent a major regulator of LN T‐cell dynamics in chronic viral infections (Ferrando‐Martinez et al., 2018; Petrovas et al., 2017). Therefore, we sought to investigate the presence of pro‐inflammatory cells in the LNs from young and old NHPs. Expression of CD68 and CD163, markers for monocytes/macrophages (Barros, Hauck, Dreyer, Kempkes, & Niedobitek, 2013), and myeloperoxidase (MPO), a marker for granulocytes/neutrophils (Klebanoff, Kettle, Rosen, Winterbourn, & Nauseef, 2013), was analyzed (Figure 5a and Figure S6a). Given the relatively lower coverage of cell size by nucleus compared to T and B cells, a factor that could affect the histocytometry analysis (Figure 5b), the quantitation of macrophages was performed using either nuclear or actin staining and cell segmentation using segmented surfaces (based on nuclear signal) or the “surface” module, respectively (Imaris). No significant difference was found between the macrophage numbers determined by nuclear or actin staining (Figure S7a). Old animals had significantly less follicular CD163^hi^ (*p* = .0142) and MPO^hi^ (*p* = .0273) cells than the young NHPs (Figure 5c and Figure S7b) while no difference was found for the CD68^hi^ cells (Figure S7c). No significant difference was found when these factors were analyzed in the T‐cell zone (TCZ) (Figure S7d). The ratio, inside the follicle to outside the follicle, of CD68^hi^ and MPO^hi^ cells was significantly higher in the young animals (*p* = .0495, *p* = .0494 for CD68^hi^ and MPO^hi^ cells, respectively) (Figure S7e). Since sequential sections were used for pro‐inflammatory and Tfh cell analysis (for comparison, see Figure S6b), we asked whether there was any association between these two groups of cells. A significant negative correlation between Tfh and CD163^hi^, CD68^hi^ cells in young animals (Figure 5d) but not in old animals was found (Figure S7f). Contrary to the follicular monocyte/macrophage profile, old animals were characterized by similar absolute numbers of circulating monocytes (Figure S8a) while significantly higher levels of circulating pro‐inflammatory mediators LPS (*p* = .0237), TNF‐α (*p* = .0297), and IL‐6 (*p* = .0431) (Figure 5e) but not IL‐17 levels were recorded (Figure S8b). Therefore, the distribution of follicular pro‐inflammatory cells is significantly different between young and old animals, a profile not readily reflected in the circulating pro‐inflammatory factors.

### 2.6 Differential impact of GC cell populations on GC B cells with aging

**Figure 6:**
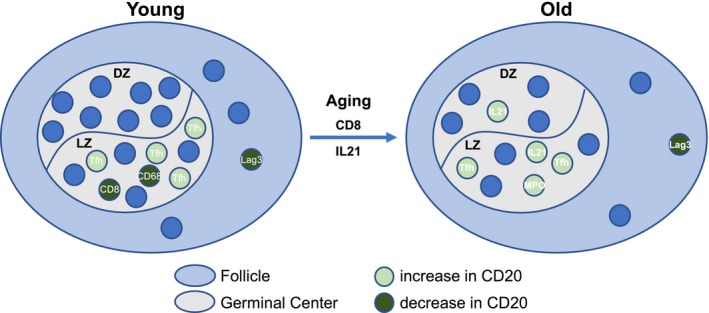
Germinal center (GC) reactivity regulation in aging. The cartoon depicts major cell populations present in the follicular areas from young and old animals. Factors (follicular CD8, IL‐21^hi^ cells) significantly affected by aging are shown too. Our data suggest a different baseline follicular cell landscape between young and aged follicles with potential impact on the regulation of GC B cells and presumably the pathogen‐/immunogen‐induced B‐cell responses

Our data show that several biological factors related to GC reactivity are affected by aging. How individual factors can affect this outcome in the context of such multiparametric processes is not well understood. We built an initial GLMM model via penalized quasi‐likelihood (PQL) with 8 cell populations (predictor; PD1^hi^CD4, IL21^hi^, CD3^hi^CD4^lo^, CD68^hi^, CD163^hi^, MPO^hi^, FoxP3^hi^, and Lag3^hi^ cells) to estimate their individual impact on CD20^hi/dim^Ki67^hi^ cells within each age group. By applying top‐down strategy, the final refined model in the young group showed significant association between PD1^hi^CD4 (*p* = .0077), CD3^hi^CD4^lo^ (*p* = .0000), CD68^hi^ (*p* = .0213), Lag3^hi^ CD4 (*p* = .0032), and CD20^hi/dim^Ki67^hi^ cells. The estimated average cell density in CD20^hi/dim^Ki67^hi^ cells increased by 0.27% with a unit change of cell density in PD1^hi^CD4 cells and decreased by 2.38% with a unit change of cell density in Lag3^hi^ CD4, 0.25% with CD68^hi^ and 0.07% with CD3^hi^CD4^lo^ cells, while holding other variables constant. A different pattern was found in the old group, where significant association was found between PD1^hi^CD4 (*p* = .0000), IL21^hi^ (*p* = .0110), MPO^hi^ (*p* = .0004), Lag3^hi^ CD4 (*p* = .0118), and CD20^hi/dim^Ki67^hi^ cells. In old animals, the estimated average cell density in CD20^hi/dim^Ki67^hi^ cells increased by 0.44% with a unit change of cell density in PD1^hi^CD4, 0.37% with MPO^hi^, 0.08% with IL21^hi^, and decreased by 0.43% with a unit change of cell density in Lag3^hi^CD4 cells, while holding other variables constant (Figure 6). Next, we built a new model to investigate the interaction of aging with 8 cell populations. By applying top‐down strategy, the final refined model revealed aging significantly affected the association between IL21 and CD20^hi/dim^Ki67^hi^ cells (*p* = .0000), CD3^hi^CD4^lo^ and CD20^hi/dim^Ki67^hi^ cells (*p* = .0005) (Figure 6). The effect of IL21 on CD20^hi/dim^Ki67^hi^ cells in the young group is 0.17% lower than in the old group. The effect of CD3^hi^CD4^lo^ on CD20^hi/dim^Ki67^hi^ cells in the young group is 0.06% lower than in the old group. Taken together, the models and data indicate that the mutual regulation between GC B cells and other GC immune cell populations may differ with respect to aging.

## 3 DISCUSSION

Investigating the effect of aging on follicular/GC immunodynamics is of great importance for the understanding of the development of pathogen‐ and vaccine‐induced B‐cell responses in the elderly. NHPs have been extensively used for the study of chronic viral infections and the development of vaccine strategies (Sui et al., 2013; Zhou, Bao, Haigwood, Persidsky, & Ho, 2013). Despite possible differences between the human and NHP lymphoid organ system, the NHP model can provide critical information for the design of new therapeutics (vaccines or interventions targeting the viral reservoir in lymphoid organs) for diseases like HIV. This model allows for the in vivo manipulation of GC immune dynamics, the study of longitudinal LN biopsies at different phases of viral infections and/or vaccinations and different anatomical sites, procedures which are impossible in the context of human clinical trials. A significant decrease of the absolute numbers of circulating lymphocytes was found in old NHPs at the steady‐state (absence of recent/current infection or vaccination) mainly affected by the selective drop of CD4 T‐cell counts. In line with previous human studies (Freitas et al., 2019), we found a shift from naïve to memory status for both CD4 and CD8 T cells in aged animals, a change more evident in blood compared to LNs. This shift was associated with an increase of the circulating “effector memory” compartment of both CD4 and CD8 T cells. Given the absence of CCR7 on effector memory circulating T cells (Farber, Yudanin, & Restifo, 2014), these cells could traffic back to secondary lymphoid organs employing alternative mechanisms like through CXCR3/CXCR3L interactions (Ferrando‐Martinez et al., 2018; Khan et al., 2000), a mechanism presumably dependent on local tissue infection and inflammation. The disconnected relative frequencies of memory CD4 and CD8 T cells in circulation and LNs imply that such recruitment of effector cells possibly does not take place in steady‐state NHP LNs. Whether this profile reflects a process favoring the trafficking of memory T cells to peripheral tissues rather than to LNs in aged NHPs is not known and needs further investigation. In addition to T cells, significant changes were found in circulating B‐cell compartments characterized by reduced frequencies of naïve and switched (IgG^hi^) and increased frequencies of memory DN B cells, consistent with reported expansion of these B cells in different settings, including aging and autoimmune diseases (Myles, Sanz, & Cancro, 2019).

We observed significant differences of GC activity between young and old NHP LNs. The dark and the light zone were largely indistinguishable in many of the old follicles, in line with studies showing that the microenvironment of the LN is disrupted with age due to disruption of the FDCs, FRCs, and other stromal cells (Thompson et al., 2017). This altered GC organization was associated with significantly lower normalized numbers of Tfh and CD20^hi^Ki67^hi^ GC B cells, suggesting disrupted mutual regulation between Tfh and GC B cells (Baumjohann et al., 2013) that could result in impaired steady‐state GC reactivity in old NHP LNs. In contrast to PD1^hi^CD4^hi^T cells, increased numbers of follicular PD1^lo^CD4^hi^T cells were found in old NHPs suggesting that possibly the differentiation of CD4 T cells to GC Tfh is affected in these animals. Circulating Tfh‐like cells could represent a small fragment of GC Tfh that exit the follicles and enter the blood. Alternatively, they could represent a LN precursor of GC Tfh that enters the circulation before further differentiation and trafficking into the GC. Contrary to LNs, we found a significant increase in circulating Tfh‐like cells in old animals that could represent altered trafficking or CD4 T‐cell differentiation in the LN. We should mention that in this study we have evaluated the dynamics of bulk relevant cell types. Whether the observed dynamics in old NHP LNs are the result of impaired differentiation or increased turnover is not known and needs further investigation.

Although the lineage origin of follicular regulatory CD4 T cells is not well understood, these cells locate within the follicle, particularly at the T‐B area border (Sayin et al., 2018), and have the capacity to control GC responses (Linterman et al., 2011). Although it was not analyzed, the increased numbers of follicular FoxP3^hi^Lag3^hi^CD4 cells in old NHPs could reflect increased numbers of circulating Treg CD4 T cells that have been shown in elderly humans (Lages et al., 2008). Our data suggest increased potential suppressor activity in the old follicles/GCs. Further studies exploring the ability of follicular Tregs to produce cytokines like TGF‐β or to suppress T‐cell activation/proliferation in young compared to old NHP LNs are necessary.

Follicular areas represent immunologically privileged areas with limited presence of potential CTLs (Connick et al., 2007). Besides their cytolytic activity, fCD8 cells can promote isotype switching of B cells in the settings of autoimmunity (Valentine et al., 2018) while IL‐6, a positive regulator of Tfh cells (Eto et al., 2011), can also induce IL21‐producing CD8 T cells with follicular helper function (Yang et al., 2016) and therefore further induce humoral responses indirectly through local IL21 production. Despite a trend for higher baseline CD3^hi^CD4^lo^ T cells in old compared to young LNs, a significant positive correlation between Tfh and CD3^hi^CD4^lo^ was found only in young animals, indicating a possible “helper” fCD8 T‐cell function in young NHPs.

Tissue inflammation and immune activation may play a significant role in follicular immunodynamics (Ferrando‐Martinez et al., 2018). As expected (Pinke et al., 2013), a higher pro‐inflammatory profile was found in the blood of old animals. In contrast, tissue analysis revealed significantly higher numbers of pro‐inflammatory cells (CD163^hi^ and MPO^hi^ cells) selectively in young follicles, a profile associated with a significant negative association between Tfh and CD163^hi^ or CD68^hi^ cells. In addition to their phagocytic function, follicular monocytes/macrophages could secrete cytokines like IL‐1β and chemokines like CXCL‐9, CXCL‐10, and ligands of CXCR3 which is highly expressed in GC T cells (Ferrando‐Martinez et al., 2018). Therefore, follicular preinflammatory cells could affect GC activity by modulating the function (Ritvo & Klatzmann, 2019) and/or local trafficking of GC T and B cells (Griffith, Sokol, & Luster, 2014). Whether these observed dynamics reflect a higher or altered function of monocyte/macrophage in young compared to old animals needs further investigation.

The development of GC immune responses is a highly regulated, multifactorial process requiring the spatial organization and orchestrated function of stromal, innate, and adaptive immunity cell types. We asked whether there is any association between the biological factors under investigation using a multivirant statistical analysis. Our data suggest differential regulation of GC reactivity between young and old animals. Interestingly, GC IL21 positivity is one of the factors mainly affected by aging, further highlighting the critical role of this cytokine, especially in old subjects. This type of analysis can identify specific cell populations/biological factors affected by the aging process, potentially providing targets for novel immune interventions aiming to strengthen the vaccine responses, especially in the elderly.

Our study revealed that (a) aging affects both Tfh and GC B cells. This profile could be due to intrinsic defects in differentiation/maintenance of particular cell types or due to a disturbed mutual regulation between Tfh and GC B cells (Baumjohann et al., 2013). Understanding the driving force(s) behind these dynamics could be informative for specific interventions (i.e., new generation adjuvants) favoring the development of specific cell populations and boosting the efficacy of vaccine candidates, (b) blocking potential suppressor mechanisms (Treg) (Swainson et al., 2019) could benefit the development of vaccine‐induced responses in aged individuals, and (c) the role of LN inflammation as a regulator of cell trafficking/interactions could differ between young and old individuals. The differential function of local monocytes/macrophages/granulocytes–neutrophils could lead to an altered chemokine network with an important effect on GC immune reactions (Griffith et al., 2014). Besides altered intratissue trafficking of CD8 T cells, the function of fCD8 cells could also differ in aging (CTL vs. helper activity), and (d) significant heterogeneity of individual follicles occurs within the same LN. The impact of such heterogeneity on immunogen/pathogen humoral responses is not known. Our previous data have shown that preservation of follicular dynamics, even in parts of the follicles of a given LN, is associated with flu‐specific responses in cART HIV‐infected individuals after vaccination with a seasonal vaccine (Moysi et al., 2018). Whether this is the case in aging too is not known and needs further investigation, and (e) an integrated analysis combining novel quantitative multiplexed imaging assays and modeling of imaging data with high dimensional phenotypic and molecular (sequencing) characterization could lead to the construction of “tissue signatures” and the identification of targets for boosting GC dynamics and vaccine responses in old individuals. Given the limited, if any, access to LN biopsies in human clinical trials, alternative strategies (fine needle aspiration—FNA) have been proposed (Cirelli et al., 2019). However, we should keep in mind that critical information (i.e., tissue structure, cell–cell positioning) cannot be obtained using FNAs, further emphasizing the importance of using the NHP model.

The described data provide an immunological basis for likely diminished B‐cell responses upon infection or after vaccination in the elderly. Although the molecular mechanism(s) behind this profile is not known, we hypothesize that it could act synergistically with other defects that occur with age, like the loss of TCR clonotyping (Gil, Yassai, Naumov, & Selin, 2015) and hyalinization/fibrosis (Hadamitzky et al., 2010; Taniguchi et al., 2003), and further diminish the mounting of effective B‐cell responses. In conclusion, many LN cell populations that are crucial for mounting an immune response are significantly impacted during aging in NHPs. Our data are consistent with previous human studies that have demonstrated a loss of organization in the GCs as well as an overall loss of B and Tfh cells (Lee et al., 2016). Further studies will explore the impact of the described steady‐state follicular dynamics in the development of efficient antiviral or vaccine‐induced B‐cell responses in aged animals.

## 4 EXPERIMENTAL PROCEDURES

### 4.1 Animals

Indian rhesus macaques were housed at the New Iberia Research Center, JHU, and the Yerkes National Primate Research Center. LNs and blood samples were obtained from each animal (Table 1). There was no history of recent/current infection or vaccination (Table 1). PBMCs and tissue‐derived cells were kept in liquid nitrogen until they were analyzed. All animals were handled in accordance with the standards of the American Association of Laboratory Animal Care, reviewed, and approved by the respective institutional review board. LN samples were fixed (4% PFA) and paraffin embedded for staining and imaging. Absolute counts were measured in whole blood by complete blood counts and phenotyping. All cells and tissues reported here were collected prior to infection or immunization.

### 4.2 Imaging studies

Images were captured using a Nikon (C2 and A1) confocal microscope operated through the NIS‐Elements AR software. A 20× (NA 0.75) dry lens and 40× (NA 1.3) oil lens were used to acquire the images—multiple fields of view and z stacks were stitched together via the NIS‐Elements AR software. Pixel density of each field of view was 512 × 512. No frame averaging or summing was used while obtaining the images. Compensation of the emitted fluorescence was achieved utilizing the NIS‐Elements AR software “live unmixing” function. Single‐stained tissues were used to generate an emission spectrum database, which was used to separate the fluorescence into the corresponding channels. Histocytometry was performed as previously reported (Petrovas et al., 2017).

### 4.3 Statistics

Histocytometry‐derived data were further analyzed using a generalized linear mixed‐effects model (GLMM) to estimate the effect of age on cell density (cell frequency/ area of follicle) in NHPs. The fixed effect quantified the effect of age group; the random effect quantified the variation across NHPs. Flow cytometry‐derived data and serum measurements were analyzed using Student's unpaired *t* test. *p* < .05 was considered as significant.

## CONFLICT OF INTEREST

The authors have declared that no conflict of interest exists.

## AUTHOR CONTRIBUTIONS

KS, SP, TS, DKK, and KBR performed the experiments, did the analysis, and reviewed the manuscript. LP performed the statistical analysis. RP and RAK provided critical help for the interpretation of the results and reviewed/edited the manuscript. LG provided material and reviewed/edited the manuscript. KS, LP, FV, SP, and CP wrote the manuscript. FV, SP, and CP conceived the study and designed the experiments.

## Data Availability

The data that support the findings of this study are available from the corresponding author upon reasonable request.
