# Supplementary figures and images for: Compromised steady‐state germinal center activity with age in nonhuman primates
Source: Aging Cell. 2019 Dec 15;19(2):e13087. doi: 10.1111/acel.13087 (PMC6996951; doi:10.1111/acel.13087)

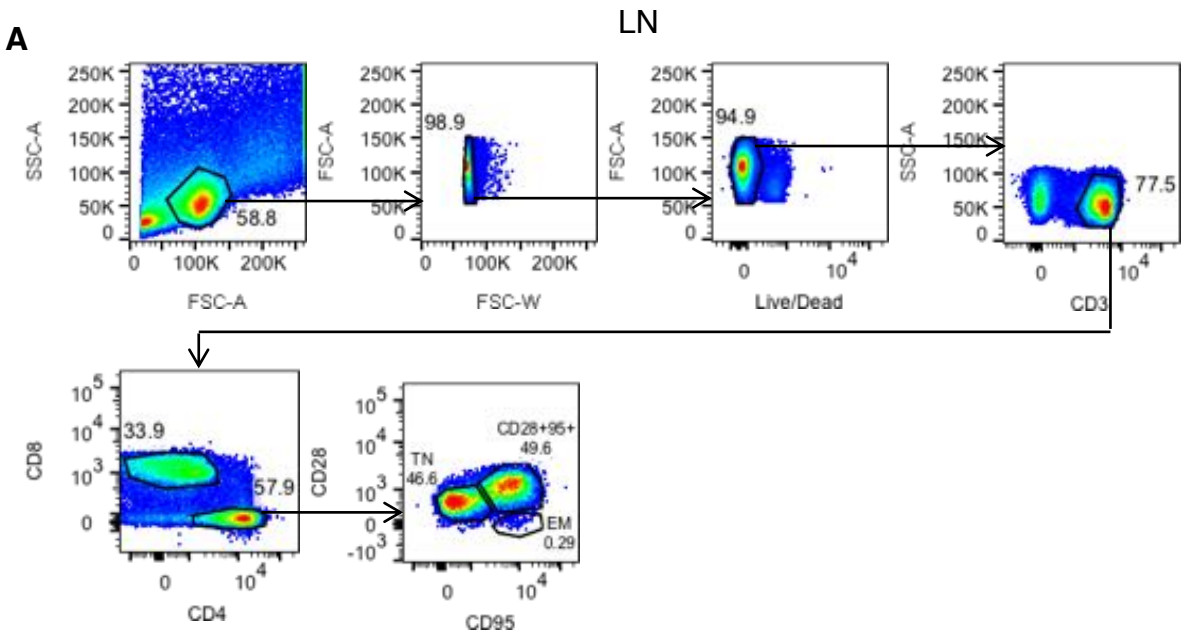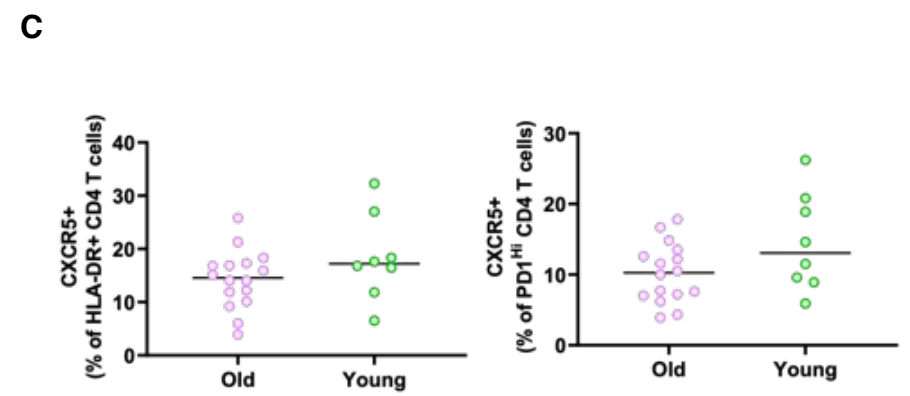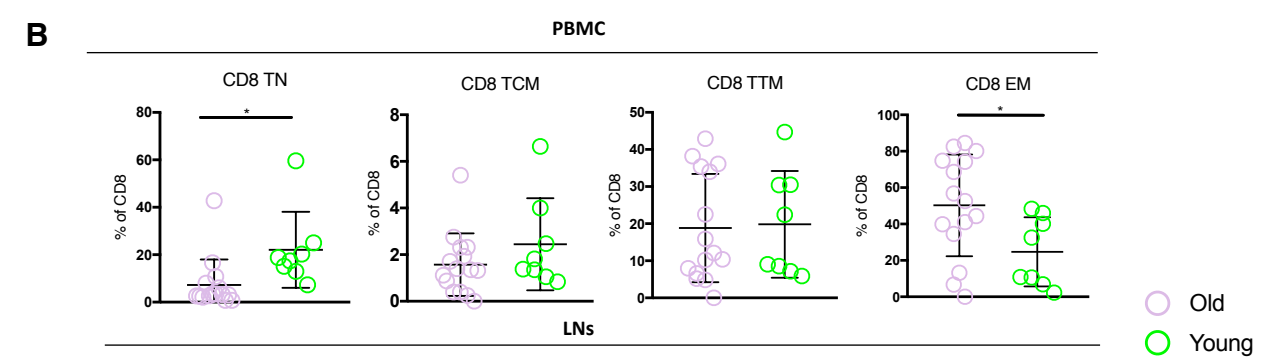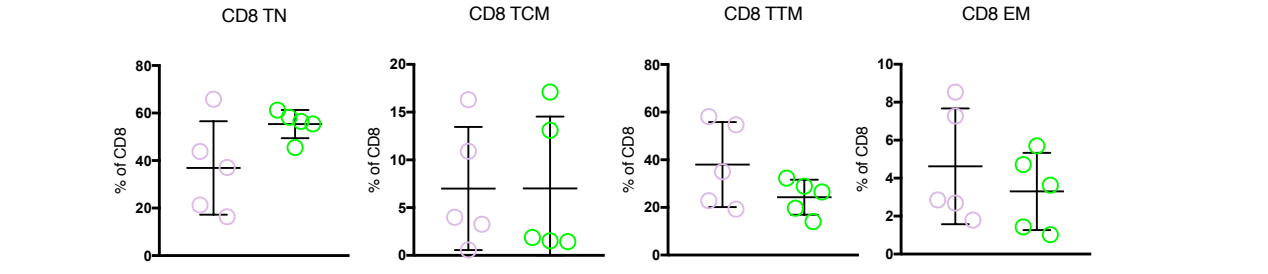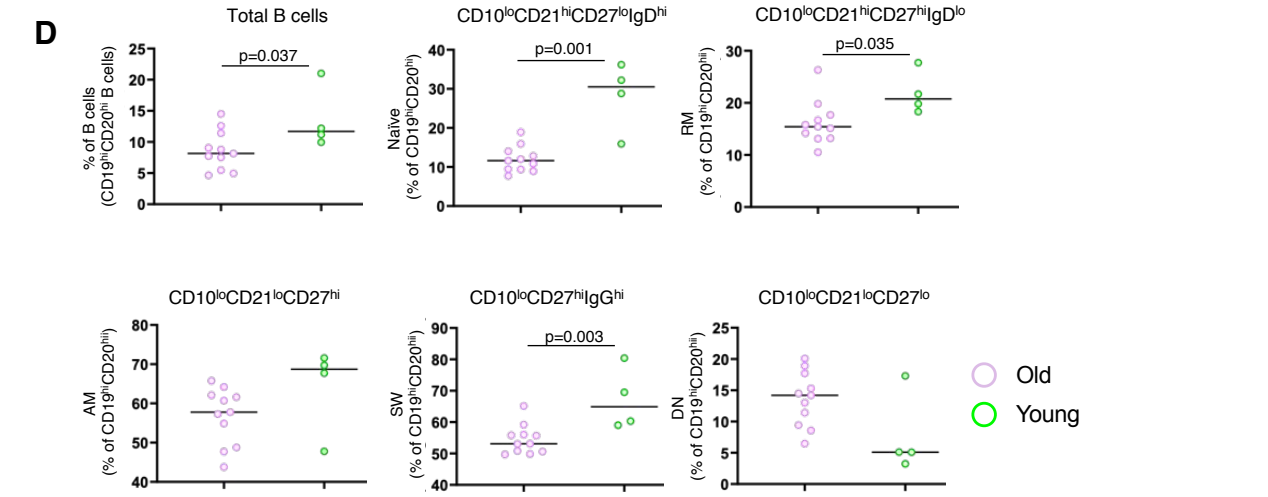

**A**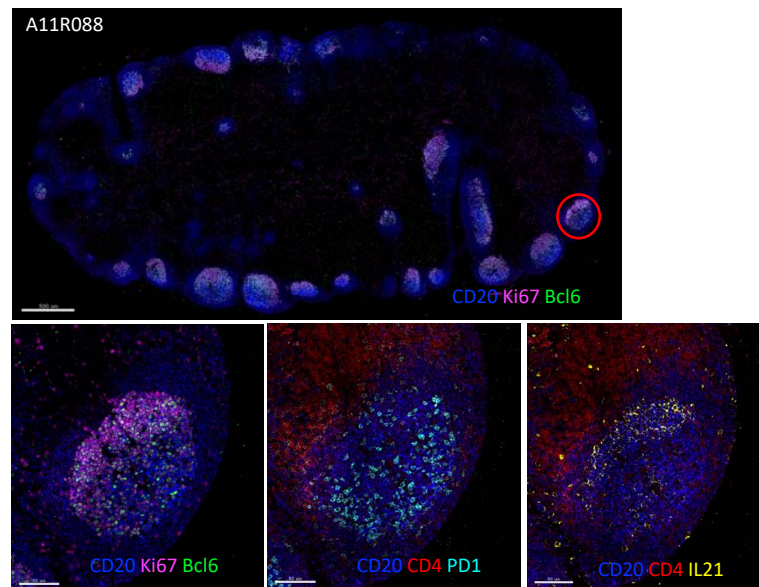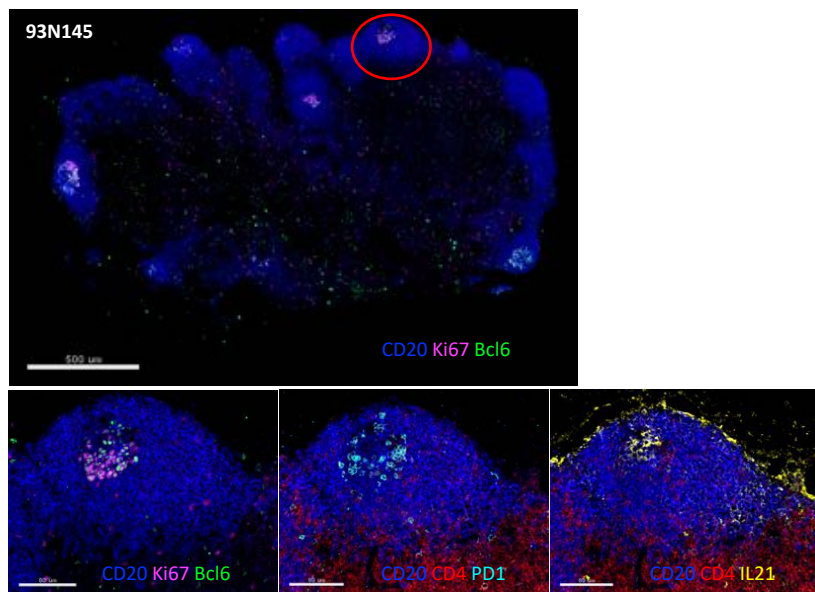**B**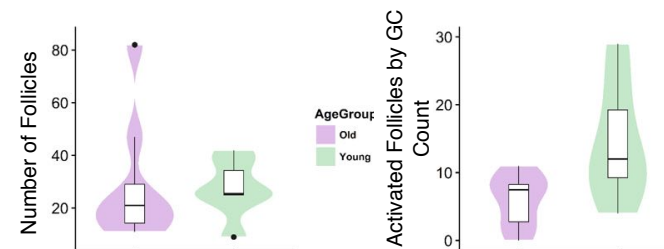**C**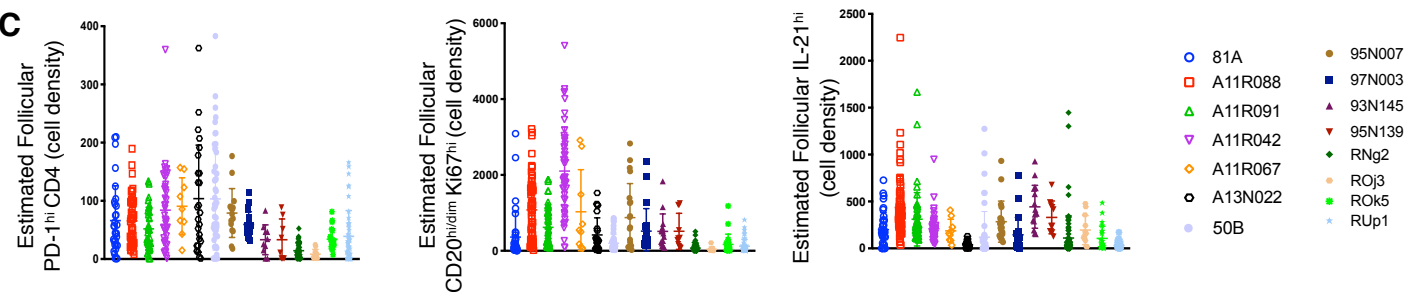**D**

LN (HistoCytometry)

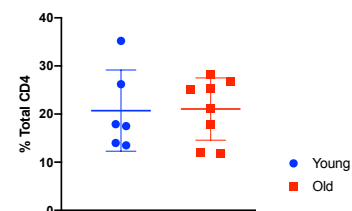**E**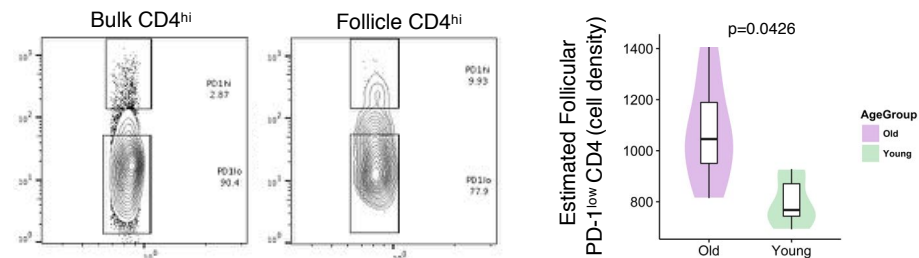**F**

LN Tfh IL-21R

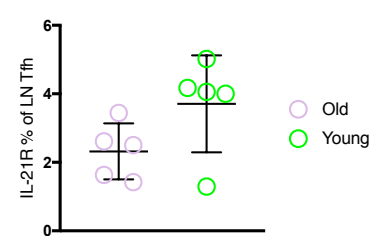**G**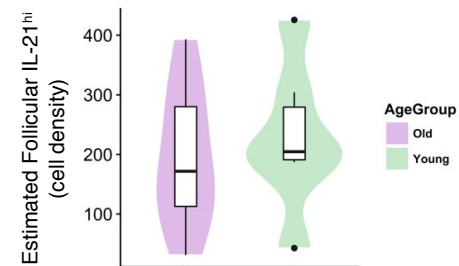

# PBMC

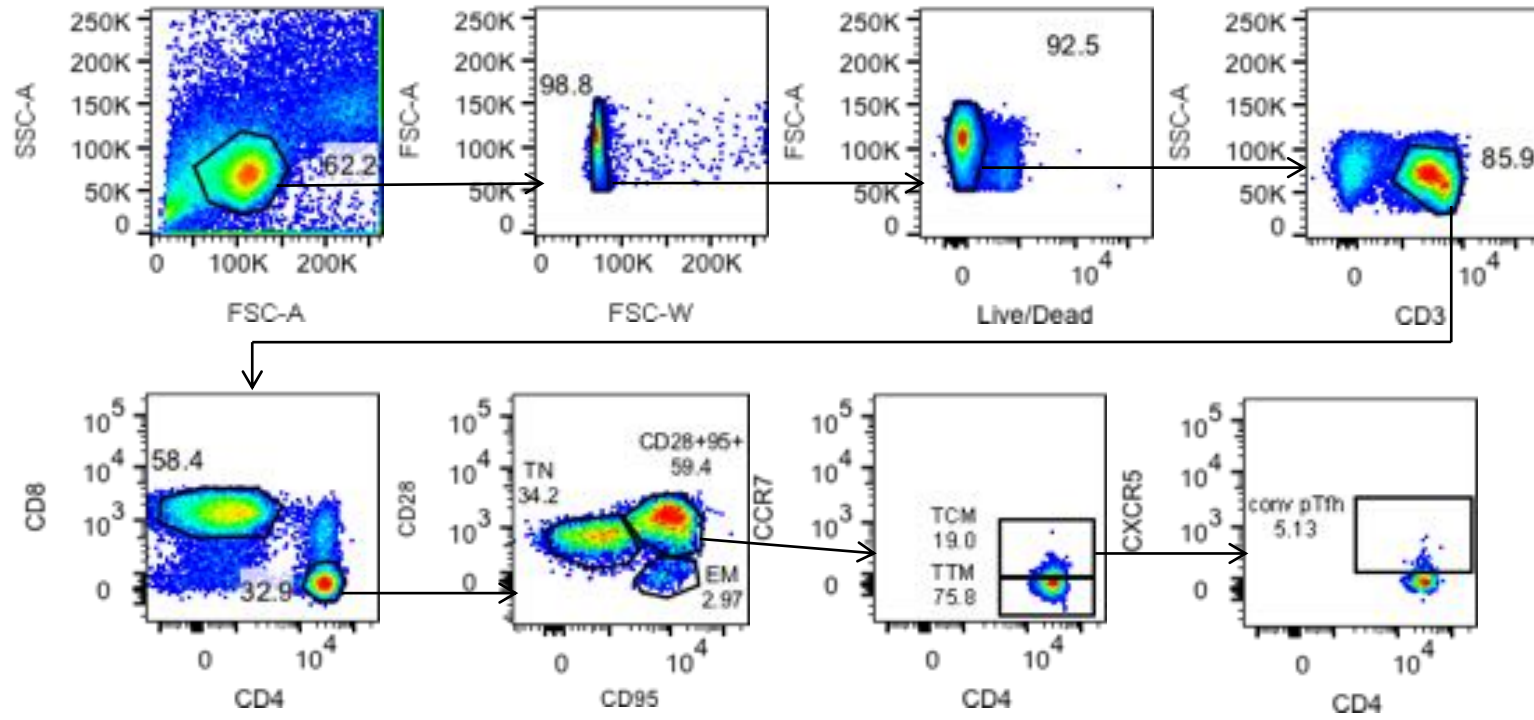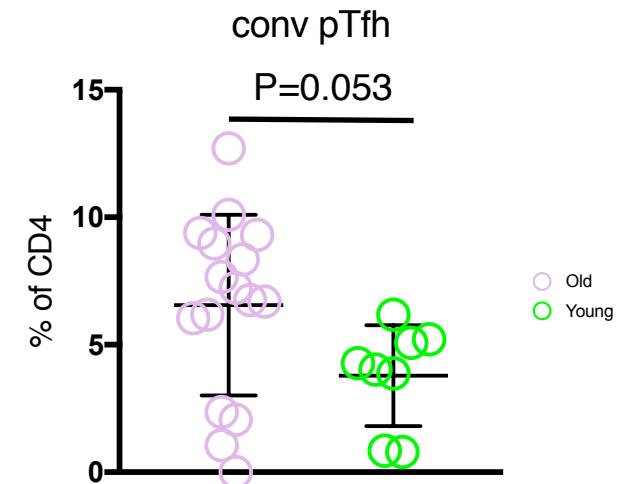

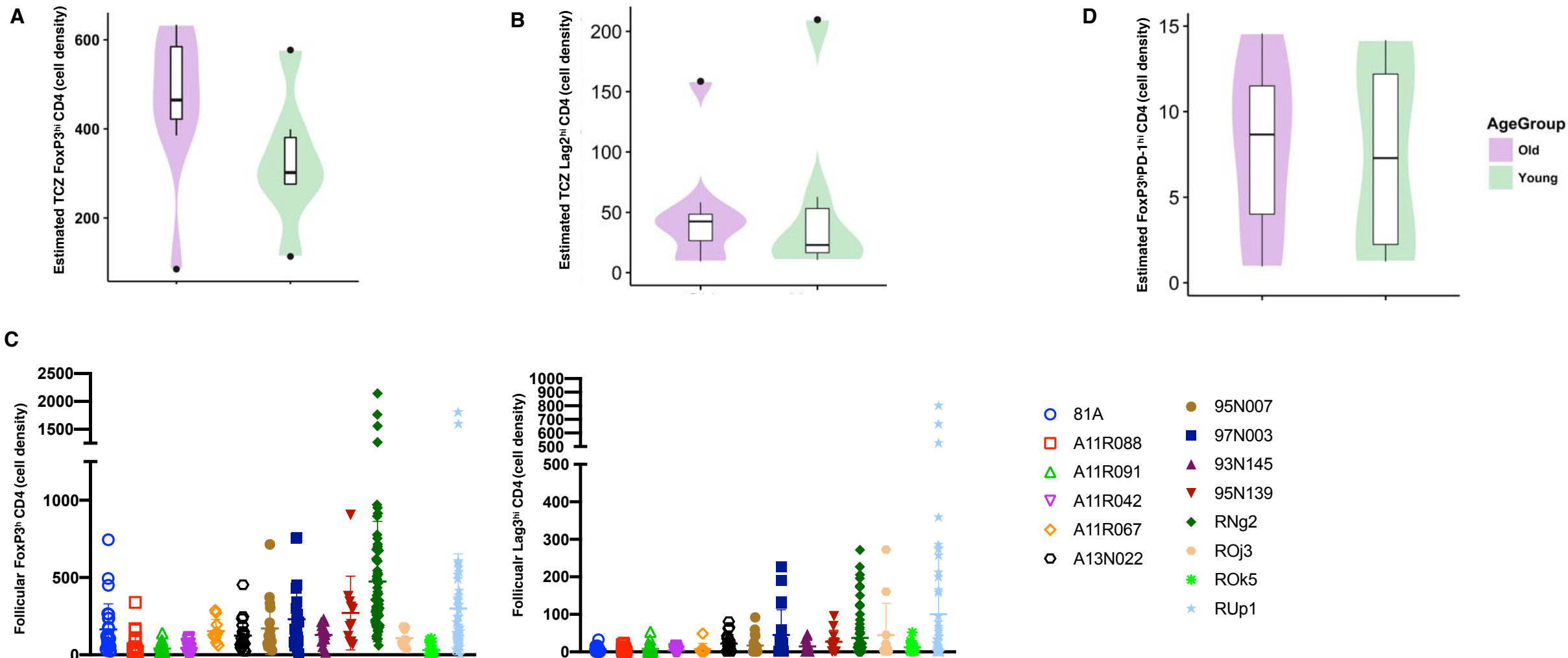

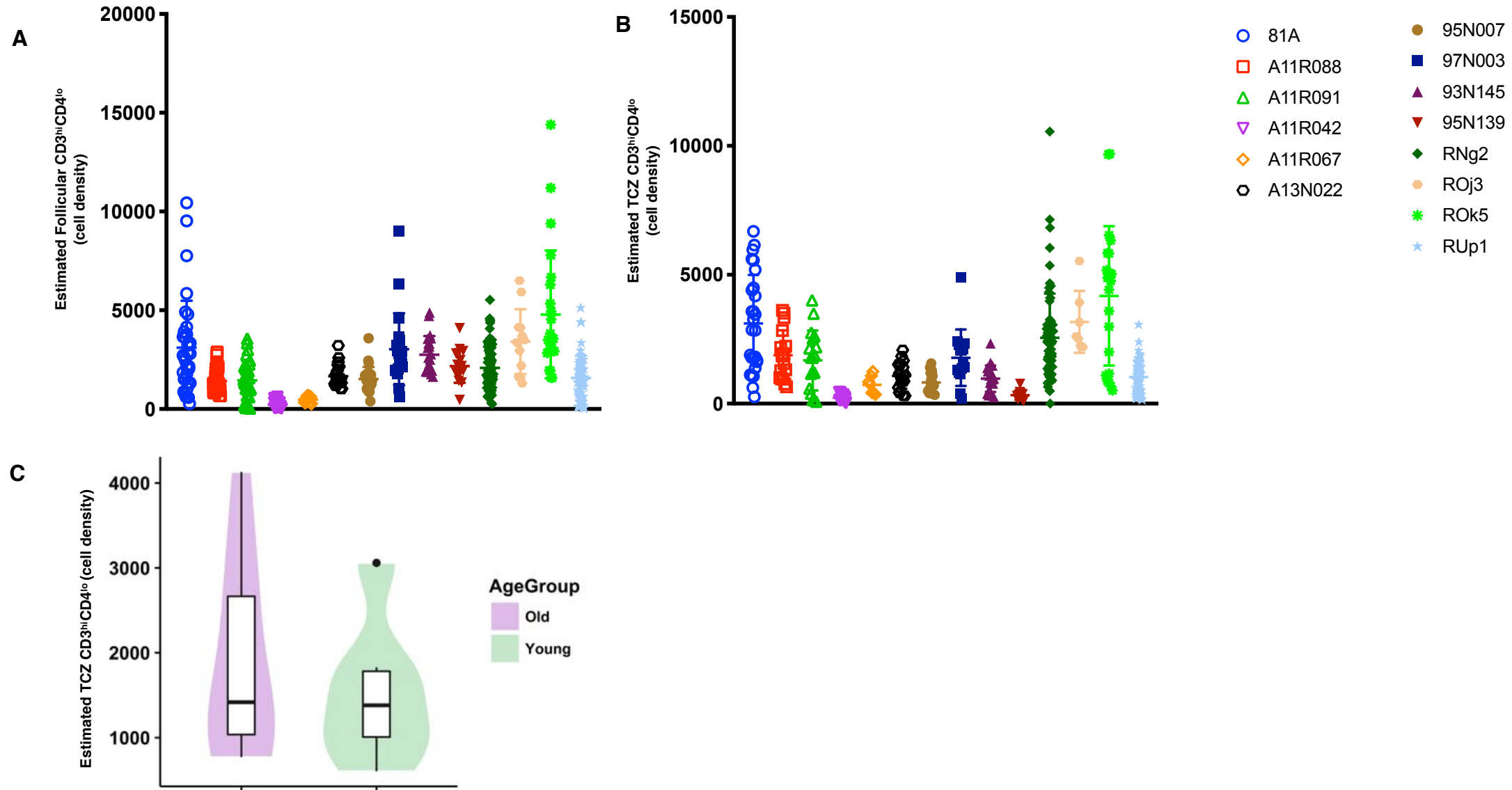

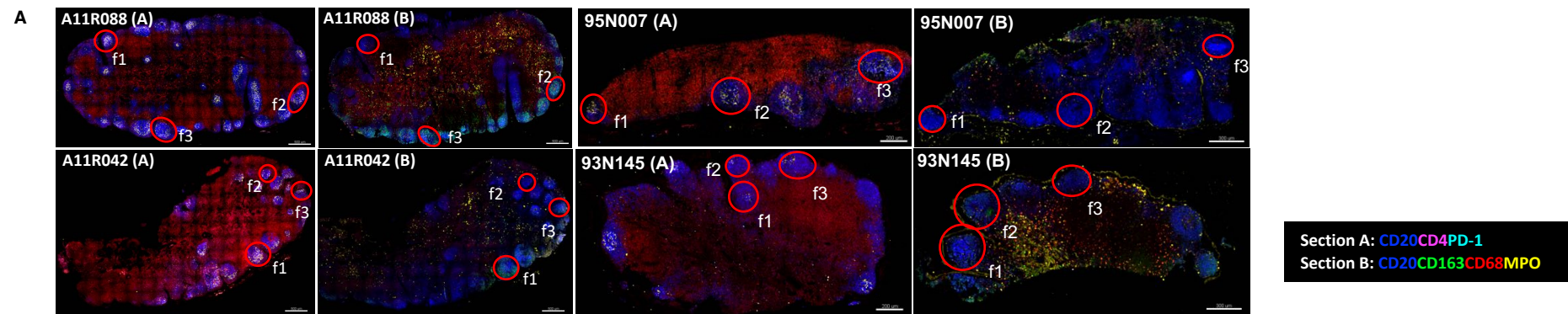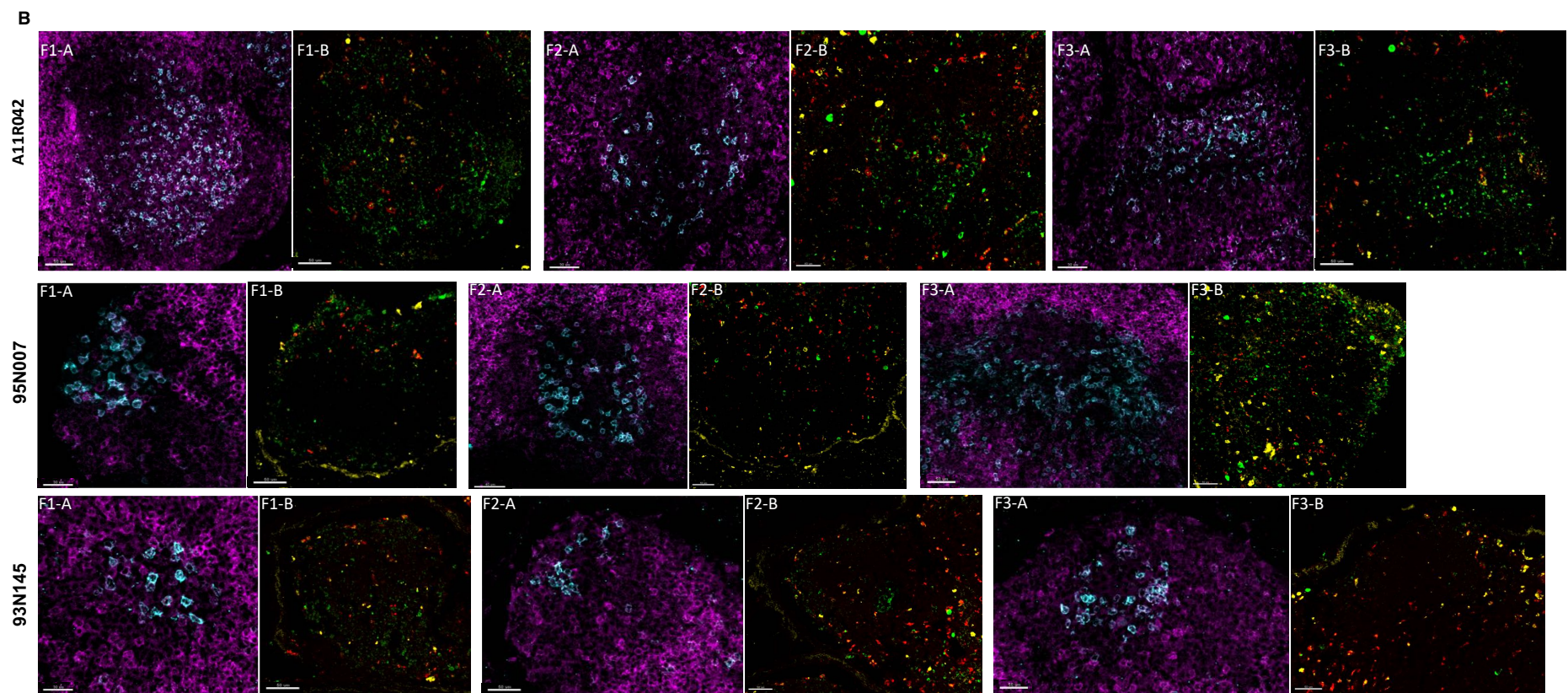

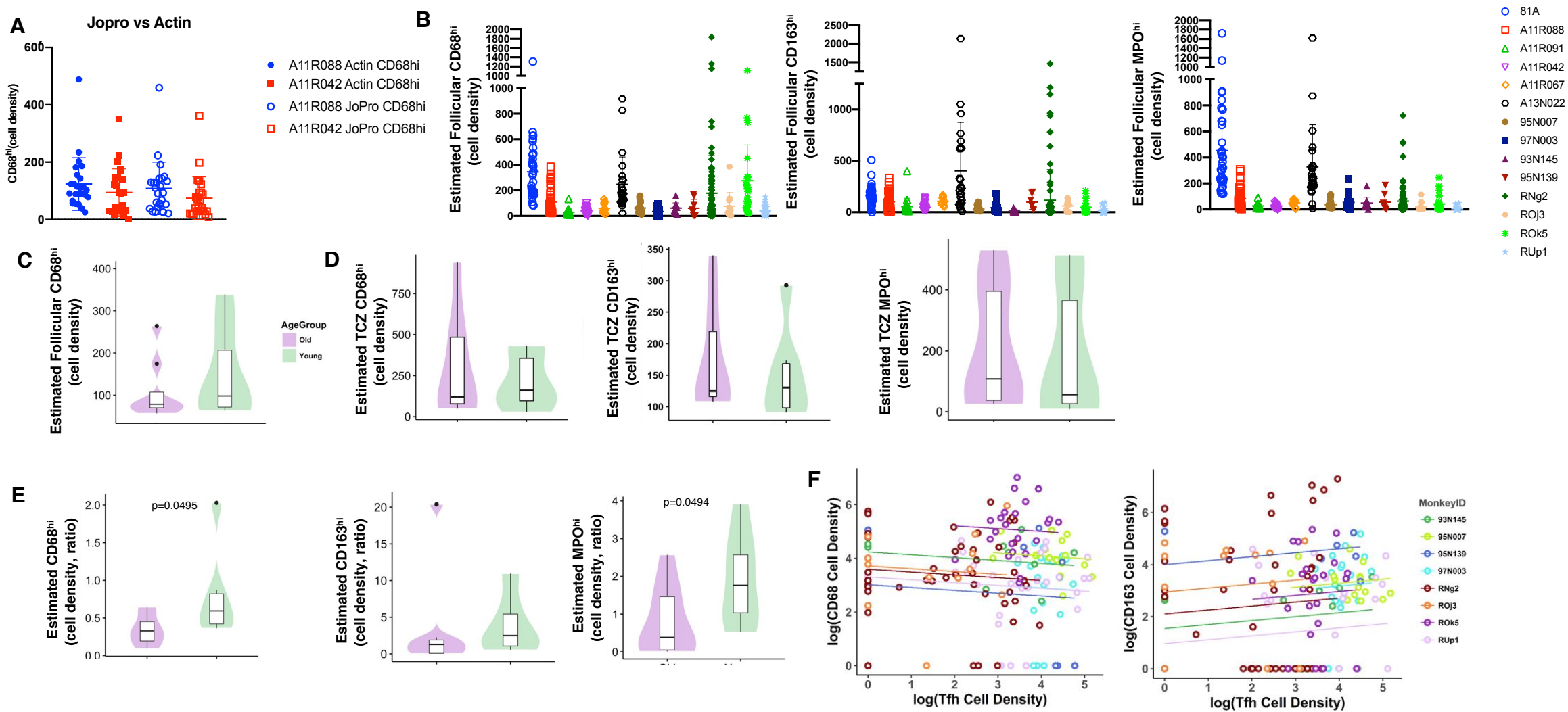

Supplement: Supplementary file 1 [file ACEL-19-e13087-s001.pdf]
