# Supplementary material for: Compromised steady‐state germinal center activity with age in nonhuman primates
Source: Aging Cell. 2019 Dec 15;19(2):e13087. doi: 10.1111/acel.13087 (PMC6996951; doi:10.1111/acel.13087)
Supplement: Supplementary file 2 [file ACEL-19-e13087-s002.docx]

**Supporting Information**

**Experimental Procedures**

**Antibodies**

*Confocal Imaging*: Primary Antibodies: Bcl-6 (Dako, PG-B6p), IL-21 (Abcam, polyclonal, Rabbit IgG), PD-1 (R&D, polyclonal, Goat IgG), CD68 (Dako, KP1), MPO (Dako, polyclonal, Rabbit IgG). PD-1 (Abcam, EPR4877), Lag3 (R&D, polyclonal, Goat IgG), CD3 (Dako, F7.2.38), IBA1(Wako, polyclonal, Rabbit IgG), Secondary Antibodies: Donkey anti-Mouse IgG Alexa 546 (ThermoFisher Scientific), Chicken x Goat IgG Alexa 647 (ThermoFisher Scientific), Donkey anti-Rabbit IgG BV421 (Biolegend), Donkey x Goat IgG Alexa 546 (Life Technologies), Goat anti-Mouse IgG1 Alexa 546 (LifeTechnologies). Primary/Conjugated: CD4 Alexa 488 (R&D, Goat Polyclonal), CD20 eFluor615 (eBio), Ki67 BV510 (Biolegend), CD163 Alexa 700 (Novus), FoxP3 (Biolegend).

*Flow cytometry:* CXCR5 FITC (Nonhuman Primate Regeant Resource, 710D82.1), CD3 PerCP-Cy5.5 (BD, SP34-2), IL-21R APC (Biolegend, 2G1-K12), HLA-DR APC-Cy7 (Biolegend, L243), CCR7 Alexa 700 (R&D Systems, 150503), PD1 BV605 (Biolegend, EH12.2H7), BTLA BV650 (BD, J168-540), CD4 BV711 (BD, L200), CD150 PE (BD, A12), CD95 PE-CF594 (BD, DX2), CD28 PE-Cy5 (Biolegend, CD28.2), ICOS PE-Cy7 (Biolegend, C398.4A), CD8 V500 (BD, SK1).

**Polychromatic flow cytometry for phenotypic analysis:** Cells were thawed and rested overnight before further use. Cells were stained with Live/Dead Blue amine viability dye (Life Technologies) and then with titrated amounts of antibodies against surface and intracellular markers. After, surface staining cells were permeabilized using BD cytofix/cytoperm solutions then stained intracellularly with Ki67. Stained cells were resuspended in 1% paraformaldehyde, acquired on a BD LSRFortessa and analyzed by FlowJo V10 (Tree Star, CA).

**Imaging Studies:** Paraffin tissue blocks were cut into 5 μm sections, deparaffinized (heating to 60^o^ C) and washed in serial baths of 100% xylene, 95% ethanol, 80% ethanol, 70% ethanol, and 100% diH_2_O. Antigen retrieval was performed (Borg decloaker, Biocare Medical, for 15 mins at 110 C in a decloaking chamber, Biocare Medical. Slides were dipped into 1X PBS to cool, permeabilized/blocked (1h, PBS/bovine albumin/Triton-X). Titrated amounts of primary antibodies were added for overnight staining (4^o^C). After washing (3 times, 15 minutes each, 1X PBS) titrated amounts of corresponding secondary antibodies were added for 2 hours/RT, followed by 3 more washes in 1X PBS. Tissues were blocked using mouse and/or goat serum (1h), followed by titrated amounts of conjugated antibodies. Following washing (x3, 1X PBS) nuclear stain (JoPro/ThermoFisher Scientific) was added for 15 min. Fluoromont G was used to mount the tissues with a glass coverslip.

**Histocytometry:** The confocal image was imported into Imaris for quantification. 3-dimensional segmented surfaces (based on nuclear signal) of spillover corrected images were generated via the Surface Creation module. After surface creation, mean intensity statistics for each marker were exported into a comma separated values (.CSV) file. The CSV file was imported into FlowJo (version 10) for further quantitative analysis (relative cell frequencies and size of imaged area).

**Plasma Cytokine analysis**

Plasma samples were thawed, vortexed, and centrifuged at 1,000 g for 3 minutes immediately prior to testing. Undiluted plasma was incubated overnight with a mixture of beads specific for IL-1β, IL-2, IL-6, IL-8, IL-10, IL-17A, IFN-γ, and TNF-α at 4°C with shaking. After washing, the beads were incubated with biotinylated detection Abs for 1 hour at room temperature. Streptavidin-PE was then added to the wells and allowed to incubate for 30 minutes at room temperature. The beads were then washed and diluted with 150 μl Sheath Fluid before acquisition on a MAGPIX instrument (Luminex Corporation). The mean fluorescence intensity (MFI) data were analyzed with MILLIPLEX Analyst Software V.3.5 (EMD Millipore). Cytokine concentrations were determined based on standard curves and expressed in pg/ml. Plasma Cytokines were determined by Magpix non-human primate cytokine magnetic bead panel (Millipore) according to manufacturer protocol.

**LPS measurement**

LPS levels were measured in plasma samples by the use of the Limulus amebocyte lysate chromogenic endpoint assay (Lonza Group Ltd) according to the manufacturer’s recommendations ([Pallikkuth et al., 2013](#_ENREF_33)). Samples were diluted 1:5 in endotoxin-free water and heat inactivated at 80°C for 10 minutes prior to the assay. LPS concentration in the samples was calculated in relation to an E. coli endotoxin standard and expressed in pg/ml.

**Data presentation**

Data showing the dynamics of relevant cell populations using histocytometry and a GLMM for their statistical analysis were presented using “violin” shapes. A violin plot is a mirrored density plot displayed in the same way as a boxplot. On each side of the boxplot is a kernel density estimation to show the distribution shape of the data. Wider sections of the violin plot represent a higher probability that members of the population will take on the given value; the skinnier sections represent a lower probability. Tails of the violins were trimmed to the range of the data.

**Statistics:**Cell frequency was estimated by adding the follicular area as an offset while fitting GLMM via negative binomial with log link by R “lme4” package. The outcome was interpreted as estimated cell frequency per unit area. Cell density was estimated by fitting GLMM via Penalized Quasi-Likelihood (PQL) with log link by R “MASS” package. For the analysis of FoxP3 subpopulations total cell density (total cell frequency / total area of follicles), the difference in the two age groups was analyzed using t-test by R “stat” package (Welch two sample t-test for FoxP3^hi^Lag3^hi^ and two sample t-test for FoxP3^hi^PD-1^hi^ population). The difference in cell density ratio (follicle/T cell zone) in 2 age groups for CD68, CD163, MPO populations was analyzed using a linear mixed-effects model (LMM) by R “lme4” package. Repeated measures correlation analysis was preformed by R “rmcorr” package between individual populations across a single follicle for young and old groups separately. In order to estimate the possible impact of individual cell populations on the outcome of the CD20^hi/dim^Ki67^hi^ population, fit data by GLMM via Penalized Quasi-Likelihood (PQL) using R “MASS” package, taking into account the random effect caused by different NHPs. Starting with 8 cell populations as fixed effect and following “Top down strategy” in young and old groups separately, we deleted non-significant fixed effect until we reached the final model. The interaction between age group and each individual population was also tested. Given that not all follicles/GCs were populated with all cell populations under investigation (total number of follicles n=408, number of follicles with complete measurements n=203), missing values were replaced with multiple imputation by chained equations using R “MICE” package. Three imputation methods, “Random Forest Prediction”, CART (classification and regression trees) and PMM (predictive mean matching) were tried and returned similar results, thus only the results from random forest imputation was presented

**Supporting Figure 1.** (A) Flow cytometry gating scheme for the identification of CD4 T cell subsets is shown. (B) The relative frequencies, determined by flow cytometry, of circulating and LN derived CD8 T cell subsets in young (8) and old (16) animals, (C) circulating CXCR5^hi^HLA-DR^hi^ or PD-1^hi^ CD4 T cells and (D) circulating total and memory B cell subsets are shown. *p<0.05 and ** p <0.01. The Student’s unpaired t test test used for the statistical analysis.

**Supporting Figure 2.** A) Confocal image (40x) of an inguinal LN from one young (A11R088, scale bar: 500 µm) and one old (93N145, scale bar: 500 µm) NHP. A magnified follicle (in red circle) from each animal is shown underneath with cell population markers (CD20-blue, Ki67-magenta, Bcl6-green, CD4-red, and IL21-yellow and PD-1-cyan. Scale bars: 80 µm). B) The absolute count of total follicles and activated follicles determined by the presence of a GC is shown in young (green, 7) and old (purple, 8) animals. A Mann-Whitney test and a Welch Two sample T test were used respectively for analysis. C) The cell density of PD-1^hi^ CD4, CD20^hi/dim^Ki67^hi^ and IL-21^hi^ cells in each follicle and animal is shown. Each dot represents a follicle. Each color/symbol representes a different animal. Open symbols: young, closed symbols: old animals. D) Relative frequencies, determined with histocytometry, of total CD4 T cells in young (blue, 6) and old (red, 8) are shown. E) Histocytometry-generated 2D plots showing the expression of PD-1 in CD4 T cells (bulk-whole tissue and a follicular area) (left panel) and the estimated cell density of follicular CD4^hi^PD-1^lo^ T cells (right panel) in young (green, 7) and old (purple, 8) animals. A negative binominal GLMM was used for the analysis. Significant (<0.05) p values are shown. F) The relative frequency, determined by flow cytometry, of LN-derived Tfh cells expressing an IL-21R^hi^ phenotype. G) The estimated cell density of the follicular IL-21^hi^ cells is shown in young (green, 6) and old (purple,8) animals.

**Supporting Figure 3.** Flow cytometry gading scheme for the identification of circulating CD4 T cell subsets. B) Dot plot showing the relative frequencies determined by flow cytometry of circulating Tfh-like CD4 T cells in young (blue, 8) and old (red, 16) animals.

**Supporting Figure 4.** The estimated cell density of T cell zone (TCZ) FoxP3^hi^ CD4 T cells (**A)** and cell density of TCZ Lag3^hi^ CD4 T cells **(B)** in young (green, 6) and old (purple (8) animals is shown. (**C)** The estimated cell density of FoxP3^hi^ (left panel) and Lag3^hi^ (right panel) CD4 T cells in each follicle and animal is shown. Each dot represents a follicle. Each color/symbol representes a different animal. Open symbols: young, closed symbols: old animals. (**D)** The cell density of FoxP3^hi^PD-1^hi^ CD4 T cells in young (green, 6) and old (purple, 8) animals is shown (two sample t-test was used for the analysis, not significant).

**Supporting Figure 5.** The cell density of follicular (A) and T cell zone (B) CD3^hi^CD4^lo^ T cells. Each dot represents a follicle. Each color/symbol representes a different animal. Open symbols: young, closed symbols: old animals. (C) The estimated cell density of T cell zone CD3^hi^CD4^lo^ T cells in young (green, 6) and old (purple, 8) animals is shown.

**Supporting Figure 6.** (A) Confocal images (40x) from sequential sections (stained with panel A or B) of inguinal LNs from 2 young (A11R088 and A11R042, scale bar: 500 µm) and 2 old (95N007 and 93N145, scale bar: 200 µm, 300 µm) NHPs. 3 follicles are circled for comparison of Tfh cells and monocytes. The panel A used to identify Tfh is CD20-blue, CD4-magenta, and PD1-cyan. The panel B used to identify pre-inflammatory cells is CD20-blue, MPO-yellow, CD68-red, and CD163-green. (B) 3 zoomed follicles (scale bars: 30 µm, 40 µm, 50 µm) from section A show the differences in distribution between the Tfh cells and pre-inflammatory cells in old and young NHPs. Follicles (scale bars: 40 µm)from the Tfh cell panel were matched to matching follicles that were stained with the monocyte panel. The Tfh are shown as CD4^hi^PD1^hi^ (CD4-magenta, PD1-cyan). The pre-inflammatory markers are MPO-yellow, CD68-red, CD163-green.

**Supporting Figure 7.** (A) Dot plot showing the CD68 cell density calculated by Histocytometry, using two different methods for cell segmentation; a nuclear (JoPro) (open symbols) and an actin based (closed symbols) one. (B) Graphs depicting the cell densities of follicular pre-inflammatory cells. Each dot represents a follicle. Each color/symbol represents a different animal. Open symbols: young, closed symbols: old animals. (C) The estimated cell density of follicular CD68^hi^ cells in young (green, 6) and old (purple, 8) animals. (D) The estimated cell densities of T cell zone CD68^hi^ (left panel), CD163^hi^ (middle panel) and MPO^hi^(right panel) cells in young (green, 6) and old (purple, 8) animals. (E) The ratio of follicular to T cell zone estimated cell densities of CD68^hi^ (left panel), CD163^hi^ (middle panel) and MPO^hi^ (right panel) cells are shown in young (green, 6) and old (purple, 8) animals. (F) The correlation between PD-1^hi^ CD4 T cells and follicular CD68^hi^ (left panel) or CD163^hi^ (right panel) cells in old animals are shown. Data from individual animals (marked with different symbol color) are shown. Each dot represents a follicle.

**Supporting Figure 8.** (A) Absolute numbers of circulating monocytes in young (blue, 4) and old (red, 4) animals are shown. (B) Levels of circulating IL-17 in young (blue, 8) and old (red, 16) animals are shown.
